# The Development and Potential of a Digital out of Home Food Environment Monitoring Platform

**DOI:** 10.3390/nu15183887

**Published:** 2023-09-06

**Authors:** Kathrin Hetz, Nuwan Weerasinghe, Holly Rippin, Kremlin Wickramasinghe, Olga Zhiteneva, Muhammad Arslan Usman, Christos Politis, Gauden Galea

**Affiliations:** 1 World Health Organization European Office for the Prevention and Control of Noncommunicable Diseases, Special Initiative on NCDs and Innovation, 2100 Copenhagen, Denmark; jayawardanan@who.int (N.W.); rippinh@who.int (H.R.); wickramasinghek@who.int (K.W.); zhitenevao@who.int (O.Z.); galeag@who.int (G.G.); 2 Faculty of Science, Engineering and Computing, Kingston University London, Kingston upon Thames, London KT1 2EE, UK; m.usman@kingston.ac.uk (M.A.U.); c.politis@kingston.ac.uk (C.P.)

**Keywords:** digital food environment, nutrition, monitoring platform, meal delivery apps, obesity, noncommunicable diseases

## Abstract

The rapidly growing field of digital meal delivery platforms has transformed the out of home (OOH) food environment, presenting both opportunities and challenges for public health. This paper introduces the development and potential of a novel digital platform designed for monitoring the OOH food environment. Drawing on publicly available data from meal delivery applications, this platform provides valuable insights into the landscape of digital food offerings, such as the most common restaurants per region, average caloric content per meal type, and energy value per monetary unit. This research addresses the current void in regulations for this digital environment, particularly around food labeling and provision of nutrition information. Even though the platform has significantly improved our understanding of the digital food ecosystem, it highlights gaps, primarily due to the lack of publicly available individual data and inconsistencies in provided information. Despite these challenges, the proposed digital platform holds considerable promise for better understanding the digital food environment, supporting healthier food choices, and informing future policy interventions aimed at regulating the online food environment. This research advocates for mandatory regulations in the digital food sector to ensure comprehensive, comparable, and transparent nutrition information and equality in access to nutritious foods.

## 1. The Issue at Hand—Unhealthy Food Environments

The burden of noncommunicable diseases (NCDs) continues to increase worldwide. In 2021, NCDs caused 90% of deaths and 85% of years lived with disability (YLDs) in the WHO European Region [1]. Obesity is a complex disease considered one of the region’s top five risk factors for NCDs. Overweight and obesity affect almost 60% of adults and nearly one in three children (29% of boys and 27% of girls) in the WHO European Region [2]. It is influenced and caused by several factors, including unhealthy food environments and dietary habits. Recent evidence suggests that around 25% of calories are consumed outside the home. This increase makes the out of home (OOH) food environment an important public health setting [3].

In most countries, meal delivery apps (MDAs) are not included in existing laws addressing establishments in the OOH food environment. This means that labeling laws and the need to disclose certain information about foods and beverages, including nutrition information, do not apply to MDAs. This results in a lack of accessible consumer information on nutritional quality of the foods they are ordering, unlike in a retail environment.

This paper will describe the development and the objectives of an innovative digital out of home food environment-monitoring platform, its benefits, goals, and challenges with a focus on the impact on nutritional information.

## 2. Digital Food Environments and Public Health

Obesogenic environments promote unhealthy lifestyles, increasing the risk of obesity and other lifestyle-related diseases. These environments make choices towards an unhealthy lifestyle, such as an unhealthy diet and lack of physical activity, easier [4]. When evaluating the OOH food environment, several factors lead to an obesogenic environment. Examples are bigger portion sizes, new opportunities to order high-calorie snacks and drinks via food delivery apps, and the increased reach of fast-food restaurants due to cheap delivery services. In addition, widespread promotional actions are increasing the popularity and awareness of OOH food products [5]. Many countries have been showing increasing consumption of out of home meals [6] and an extensive calorie intake when eating outside the home [7,8,9].

The obesogenic environment has stretched from the physical environment to the virtual world, making it a growing public health challenge. Online activities and entertainment offers for people of all ages have increased, and targeted and personalized marketing strategies make marketing even more persuasive. Also, diet-related misinformation found online can have a dangerous impact on people’s health [10]. Extensive options to purchase food or ready meals online to be delivered directly to the consumer’s house support a sedentary lifestyle and the tendency to choose nutrient-poor and high-energy foods [11].

This trend highlights the importance of understanding and regulating the digital food environment to ensure the availability of nutritious meals contributing to a healthy diet. To support this, the WHO European Office for the Prevention and Control of Noncommunicable Diseases (NCD Office) has started several workstreams exploring the digital food environment, its challenges, and opportunities. This includes a number of factsheets and reports which explore a systems approach to MDAs and describe the need for comprehensive data and a detailed understanding of MDAs to facilitate healthier choices [3,11,12,13]. MDAs are online platforms allowing people to order ready meals and beverages online via restaurant accounts, and delivery workers bring the food to the consumer. This form of food delivery has gained popularity, especially during the COVID-19 pandemic. A recent study looking at the healthiness of food and menu items in three cities in in New Zealand showed that over 75% of food and menu items offered on MDAs were classified as unhealthy based on Eating and Activity Guidelines for New Zealand Adults [14]. This trend has been observed in previous studies [15,16,17].

### 2.1. The Importance of Access to Nutritional Information

Nutrition labeling is essential to inform people better and enable them to make healthier food choices. How information is presented and the amount of information differ across countries and depend on the type of labeling—front of pack labeling (FOPL) or back of pack labeling. The WHO “best buys” recommend FOPL as cost-effective way to tackle NCDs and to support consumers in making healthier food choices [12].

In addition to improving the healthiness of consumers’ food purchases, nutrition labeling can be used for several measures that can support populations’ diets through policy decisions. Regarding marketing regulations, nutrient profile models (NPMs) with clear thresholds can indicate what types of foods are categorized as permitted or not permitted to be marketed in certain settings. Some forms of labeling might also encourage companies to reformulate their products into healthier versions to obtain a better label which might be more attractive to consumers [12].

In the European Union (EU), providing a nutrition declaration on the back of packs of food products is mandatory [18]. However, such regulations typically only apply to the physical food environment, leaving the online food environment, including MDAs, widely unregulated, which leads to a need for nutritional information for food products when ordering online.

Some larger international food chains provide nutritional information for their products on their brand websites. However, this information needs to be presented in a more user-friendly and consistent way, as the current presentation makes it hard for consumers to compare products and make healthier choices. Additionally, nutritional information is rarely provided when ordering food through MDAs, which is becoming a common way to purchase ready meals.

Research shows that meals delivered through MDAs have larger portion sizes and are higher in energy, sugars, salt, and fats compared to meals cooked at home or pre-packed meals purchased from a retailer [19,20]. The easy access to alcoholic beverages, which are also promoted on MDAs, makes alcohol purchases easier and increases associated health risks [21]. Data gaps and lack of regulation around MDAs make it challenging to monitor and understand the health risks of the digital food environment and hard to ensure access to safe and healthy food. Several considerations, including food supply chains, availability and accessibility, prices and affordability, and marketing, regulation, and desirability of food environments, must be considered [19,22]. A systems approach is needed to sufficiently understand the challenges and opportunities of MDAs.

### 2.2. The Beginnings of Change?—Calorie Labeling in a WHO Euro Member State—The UK Case

In April 2022, the UK became the first country in the WHO European Region to introduce a new mandatory regulation for the provision of calorie information on foods and beverages in cafes, restaurants, and takeaways with more than 250 employees. The display of calories does not only include packaged foods but also nonprepacked foods and soft drinks. “Calorie information must be displayed on menus, online menus, third party apps, food delivery platforms, and food labels at the point a customer is making their food and drink choices.” [23]. Further, daily recommended calories need to be added next to the calorie information of food items, menus, and labels. This new regulation is a step towards supporting customers to make healthy and informed food choices. It is part of a broader strategy to tackle obesity in the country, as research indicates that 20–25% of adult calorie intake in the UK happens outside the home [23].

Although some data on the out of home food environment have been collected, it needs to be carried out systematically otherwise they have only limited value. Further, no regular monitoring of the existing data has been conducted. Therefore, accurate, accessible, and consistent data for the region are needed in order to assess the digital OOH food environment and utilize opportunities to improve people’s access to healthier food choices.

Previous efforts have focused on the collection of data from big chains only [24,25,26]. The platform which was developed by the WHO NCD Office will support a better understanding of the digital food environment by collecting relevant data including all available nutrition-related information from MDAs, but also prices, cuisine types, and delivery areas.

## 3. Project Description of WHO/Europe OOH Dashboard

### 3.1. Aims, Function, and Opportunities of the Dashboard

The OOH food environment platform was developed in 2021 by the WHO NCD Office in collaboration with Kingston University, United Kingdom, to work towards the current lack of online information in terms of the nutritional value of foods in MDAs and fill current gaps.

The platform currently collects data from two major food delivery platforms and provides information on available restaurants, their geographical location and delivery reach, as well as menu items if available. However, most restaurants do not provide information on nutrient composition.

A technical paper describing the details of the data-monitoring platform will be published separately.

Labeling laws and regulations on the provision of nutrient information on nutritional composition values enable access to relevant food-related information in the physical food environment, including sales data and information on consumer behaviors [8,11]. The same level of data describing the digital food environment on OOH food platforms should be made available in a clear and consistent manner. Available data are essential to successfully develop and implement policies that can monitor the digital OOH food environment and improve health outcomes.

Currently, the data platform collects data from Ireland, Italy, Slovakia, Spain, and the United Kingdom. Restaurant information, including location, offered cuisine types, delivery prices, delivery area, and images, when available, was collected from each country. With more data collected by the platform, more impactful comparisons are possible. Therefore, countries are encouraged to use the OOH data platform.

### 3.2. Current Gaps: Nutrition Information in the European Context, Challenges of the OOH Environment

There needs to be more research into how food delivery apps are structured, which menu items are placed in prime positions at the top of the page, and the use of promotions and bundles to encourage the consumer to buy more. Such sales promotions can lead to excess energy consumption and overweight and obesity. However, these data points are owned by food delivery apps and are not accessible to public health researchers.

Information on food sales and food retail data can be used by policymakers in multiple ways, for example, using the data for introducing policies, including product-related, price-related, promotion-related, and place-related policies, and monitoring adherence to national dietary guidelines [22].

To develop and implement policies, policymakers need to understand in their own country what are the main contributors to the population level of salt intake or sugar intake. To estimate that, one needs the specific nutrient value per product and its sales data in a country.

Promotional offers and sales in the OOH online food environment take a lot of work to monitor. The newly developed WHO OOH-monitoring platform can help understanding the offered food items and online restaurant menus of food delivery apps, the structure of food offers and listing of items, offered menus and bundles for a reduced price, promotional events, cost of products, and the number and geographical location and reach of restaurants, which is essential from a public health perspective to better understand online food retail, including the type of food which is purchased most often, comparing geographical areas and socio-economic averages with food products. These analyses can support the implementation of regulations to protect vulnerable populations.

Some information is currently not provided on MDAs and can therefore not be collected, namely the nutritional content of food items, any kind of individual customer data like purchase behaviors or types of customers, as well as marketing strategies. Other data, including individuals’ sales data, are only accessible to the MDAs themselves.

### 3.3. Development and Structure of the Dashboard

**Figure 1:**
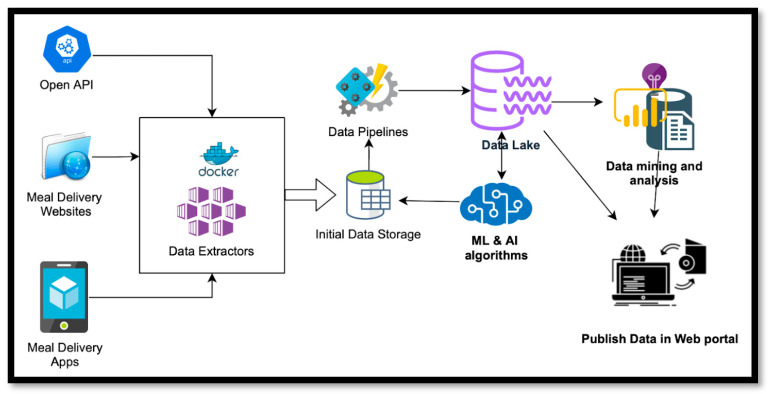
Development and structure of the dashboard.

Figure 1 describes the implementation of an MDA data-monitoring platform in the Microsoft Azure cloud platform. The platform includes base data extractors designed to extract data from multiple sources, such as open APIs, websites, and mobile apps. These extractors were extended for each MDA platform by modifying them to suit the unique features of each MDA. Subsequently, data extractors were configured for each county and installed as docker containers, with collected data initially stored in Azure storage. Several data pipelines were developed using Azure functions to process initial data cleansing and upload data to the data lake. Although the collected data formed the basic version of the data lake, there were duplicates and incomplete information, and thus data cleansing was performed to make them available to stakeholders. While traditional data cleansing methods provided a baseline for developing the data cleansing framework, they could not effectively deal with large amounts of data without incorporating AI and machine learning techniques.

**Figure 2:**
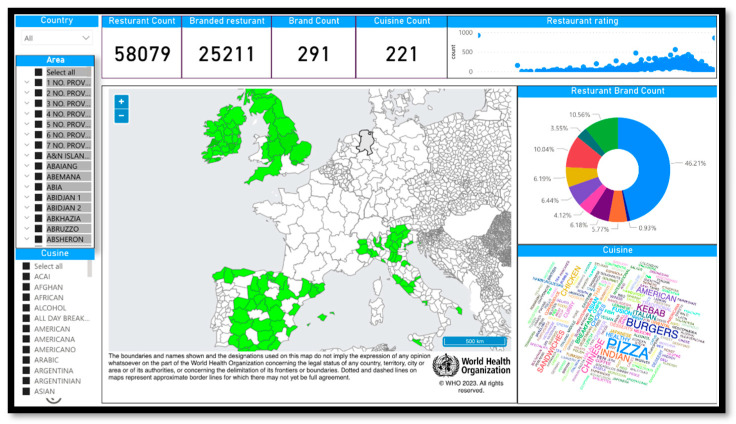
Dashboard.

Consequently, ML and AI algorithms were employed to identify the same restaurant on different platforms. Although various data cleansing approaches employing AI and machine learning techniques are available, data cleansing remains challenging and requires further research. Finally, a data mining service was developed for initial data analysis, with results stored in a Microsoft SQL database. A PowerBI-based dashboard was created to visualize and share results with policymakers, providing insights into customer behavior, popular restaurants, and trends in the meal delivery industry. Figure 2 shows a screenshot of the dashboard.

#### First Results of the OOH Dashboard

The digital OOH food environment-monitoring platform is currently able to extract and use publicly available data from MDAs and fill knowledge gaps on the OOH food environment. Important questions like the most common restaurants in a country/region, the delivery reach per restaurant, or the number of restaurant types per 100,000 inhabitants per region can be assessed. A comparative analysis can be conducted when looking at the calorie information (when provided). Average calories per restaurant/restaurant type or food type can be calculated, and meal types can be compared between restaurants/restaurant types. This will not only help to better understand the digital food environment, including the nutritional value of foods restaurants provide through MDAs, and help consumers to make informed decisions and have the opportunities to compare several dishes and food options but also indicate where policy interventions like labeling policies could support healthier food choices for people using MDAs. However, more online nutrition information is needed to compare the nutritional quality of restaurants when using MDAs. Also, positive nutrients like fiber would be beneficial in assessing the contribution to a healthier diet.

Even though the data platform already contributes greatly to a better understanding of the digital food ecosystem, there are still gaps. MDA platforms do not share individual data, meaning studies on user behavior and consumer data cannot be conducted. This information would be needed to understand how MDAs are used, how certain marketing or promotion strategies work, and how consumer behavior can be influenced to encourage healthier behaviors. Even data analysis with generally available data can be difficult due to missing menu items and the different ways in which they are presented. The biggest problem is the lack of available data in general. As the first country to make information on calories mandatory, the UK set the first step in the right direction. However, more must be done to understand, analyze, and regulate the digital food environment to support populations in having a healthy diet and maintaining a healthy weight. As in the offline food environment, the online food environment needs policies to make regulations mandatory and create a level playing field.

### 3.4. Example Analyses

**Figure 3:**
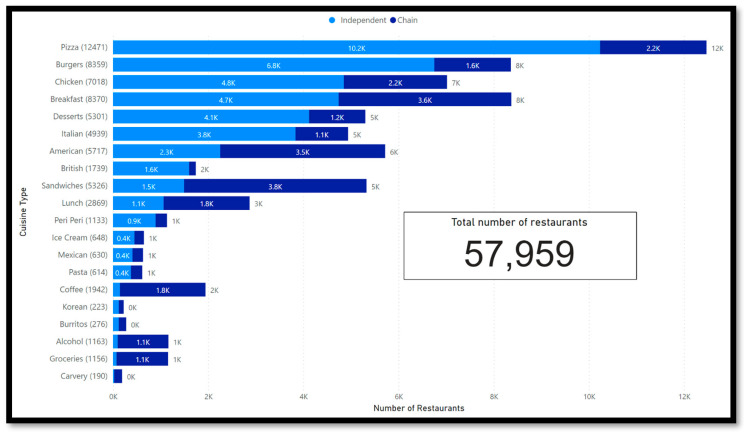
Number of Chains vs. independent Restaurants by Cuisine type.

Figure 3 shows that pizza places are the most common cuisine type on the MDAs, with nearly 12,500 restaurants. Pizza, burgers, and breakfast are the cuisine types with the most restaurants.

When comparing the number of chains versus independent restaurants by cuisine type, as presented in Figure 3, independent restaurants dominate the market on MDAs. Only the categories American cuisine, sandwiches, lunch, coffee, alcohol, groceries, burritos, and carvery have a higher number of chain restaurants. The current UK regulation demands restaurants with more than 250 employees to disclose calorie information. Therefore, only larger businesses are affected. The authors believe more research is needed on the impact of alcohol and grocery delivery of prepared foods.

**Figure 4:**
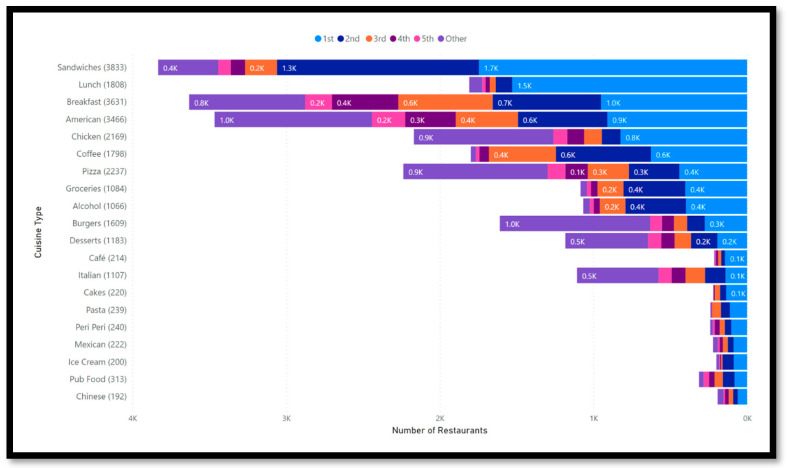
Number of Chain Restaurants by Cuisine type.

Looking closer at the main chain restaurants by cuisine type, Figure 4 shows that the five most common chains often make up more than half of the restaurants of one cuisine type. These big chains can therefore influence the online food environment widely. For example, looking at the category sandwiches, the first and second largest chains make up around ¾ of all sandwich restaurants. For lunch places, this trend is even more prominent. For example, 83% of restaurants in the category lunch are from the same chain.

**Figure 5:**
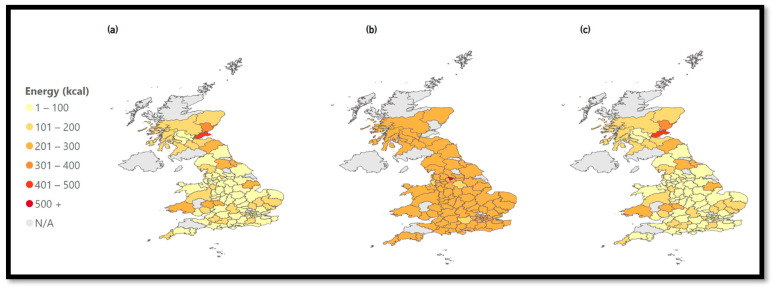
Energy density of chains. (**a**) Energy—All Chains, (**b**) Energy—Top 3 Chains, (**c**) Energy—Except Top 3 Chains.

The data platform can calculate average calorie information per cuisine type. Figure 5 compares the energy density of all chain restaurants with the top three chains and all chains except for the top three chains. The top three chains have the highest energy density, with an average of 201–300 kcal per dish.

The energy density of all food options available on the MDAs can be compared. Figure 5a shows all chain restaurants. The north of England, Scotland, and Wales seem to have slightly higher average energy values than the rest of the UK.

A substantial change can be observed when filtering for the top three chains in the UK (Figure 5b). The map changes color throughout the entire UK with average energy values between 301 and 400 kcal. This means that the most popular chains available on MDAs have higher average energy values compared to the rest.

In Figure 5c, all chains except for the three most common chains show similar average energy values (201–300 kcal) throughout the UK with three exception areas.

The high prevalence of chain restaurants impacts the average energy density.

**Figure 6:**
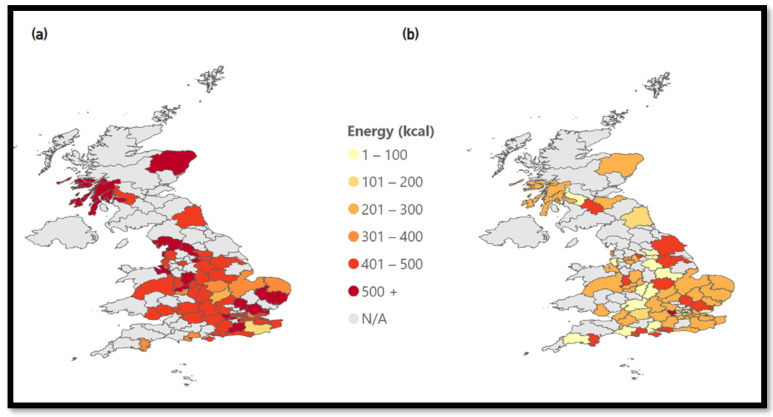
Energy of burger chains. (**a**) Burgers: Energy—Top 3 Chains, (**b**) Burgers: Energy—Except Top 3 Chains.

This trend can also be observed when only taking burger chains, the second most common cuisine type, into account. Figure 6 shows that the three most popular chains have, on average, higher energy values than less popular chains, with the most popular clearly dominating the market.

**Figure 7:**
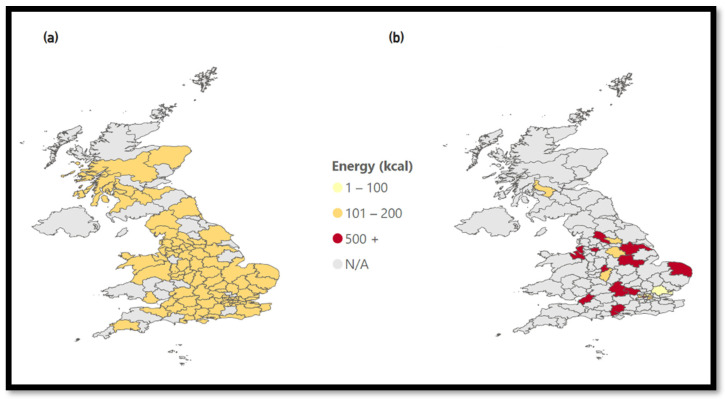
Energy of coffee places. (**a**) Coffee: Energy—Top 3 Chains, (**b**) Coffee: Energy—Except Top 3 Chains.

Coffee places have a large distribution throughout England and Scotland, mainly driven by the three largest chains. Energy data from coffee places differ from the trend of burger restaurants. Figure 7 shows that while the top three chains reach all of the UK and have average energy values from 101–200 kcal, the average energy of foodstuffs from coffee places except the top three chains is a lot higher at over 500 kcal, but the reach is mainly in the south and middle of England. One possible explanation might be the significant presence of big coffee chains, while small bakeries or coffee places might have a smaller menu.

**Figure 8:**
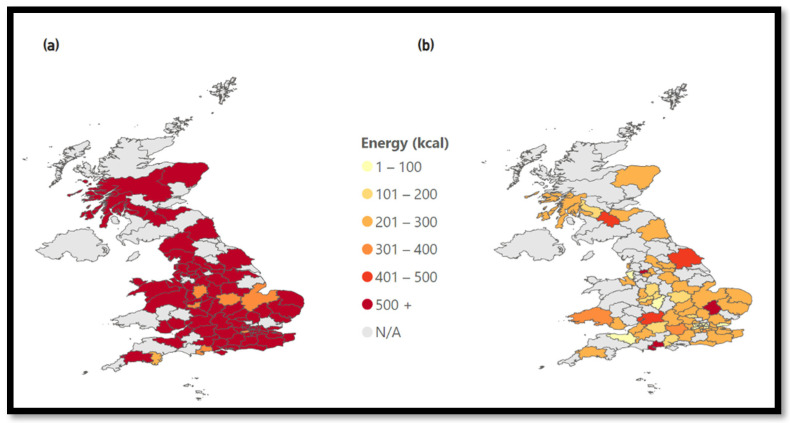
Energy of chicken chains. (**a**) Chicken: Energy—Top 3 Chains, (**b**) Chicken: Energy—Except Top 3 Chains.

Chicken restaurants have a high coverage throughout the entire UK. Chicken cuisine is dominated by popular chain restaurants, as presented in Figure 8. The top three chains sell products that, on average, have energy values of over 500 kcal and cover nearly the entire UK. At the same time, the remaining restaurants have lower average energy values and also a smaller reach.

**Figure 9:**
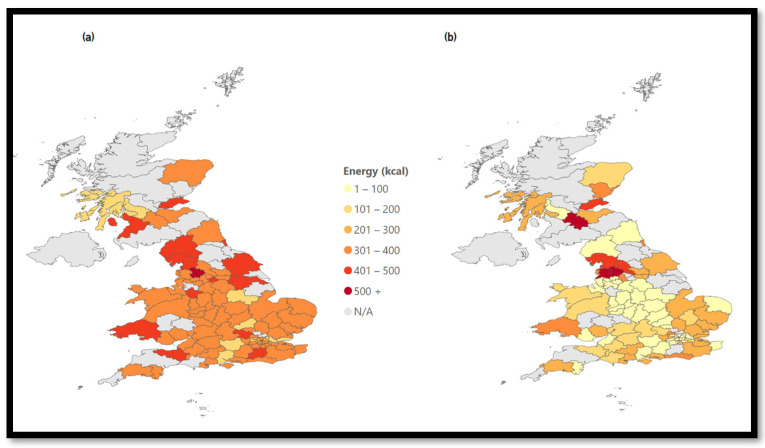
Energy of pizza chains. (**a**) Pizza: Energy—Top 3 Chains, (**b**) Pizza: Energy—Except Top 3 Chains.

Figure 9 shows that pizza places have the same tendencies. While the top three chains have a high reach and higher average energy values, mainly ranging from 301–400 kcal, the less common chains tend to have foods with lower average energy counts.

**Figure 10:**
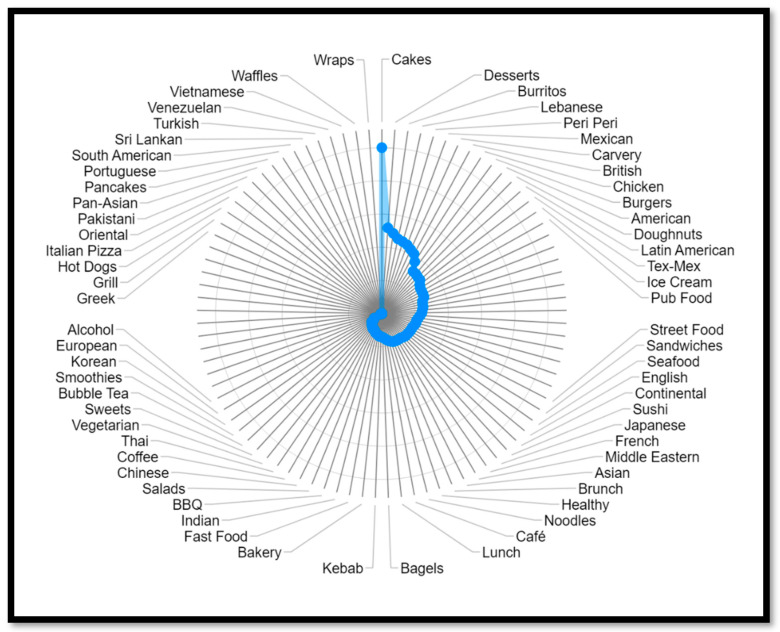
Average energy by cuisine type.

Comparing cuisine types by their average energy value, as presented in Figure 10, helps to increase the understanding of the OOH food environment. When comparing average energy values by different cuisine types, which products contribute the most to the average value, how large the product variety is, and how the food contributes to the daily energy intake should be considered.

**Figure 11:**
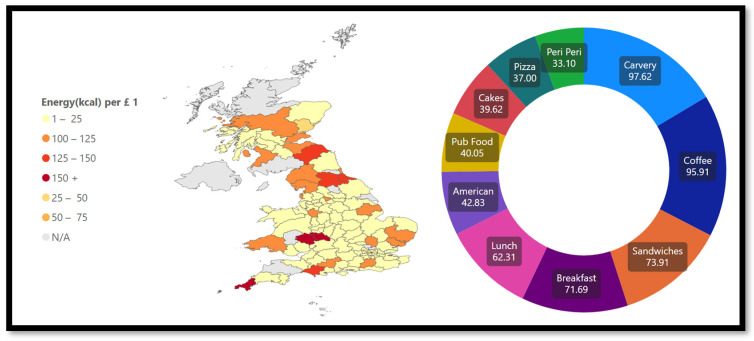
Energy per GBP 1.

When analyzing the OOH environment, energy (in kcal) per monetary unit is an important factor to consider. This is essential when discussing equality and access to healthy and nutritious foods in deprived areas. Figure 11 shows that the categories carvery (98 kcal per GBP 1), coffee (96 kcal per GBP 1), and sandwiches (74 kcal per GBP 1) have the highest amount of kcal available for GBP 1, while peri peri (33 kcal per GBP 1), pizza (37 kcal per GBP 1), and cakes (40 kcal per GBP 1) are on the lower end. Again, the variety of products and a list of the most popular products are necessary to understand consumer behavior. Appendix A shows a list of cuisine types including their average and maximum energy per menu item/kcal. It also indicates number of chains per cuisine type.

## 4. Discussion

Food products that are sold in the physical food environment are under stricter regulations in terms of food safety and nutrition declarations compared to the online food environment. Health authorities should not only control food safety but also the healthiness of the food offered in this fast-growing online sector. This is difficult due to missing data like consistent and comparable nutrition information of offered foods like those offered on the back of pack label in the offline food environment, consumer-specific information to understand purchase behaviors of customers, and comparison and analysis of the restaurants offering food online. Without a uniform labeling system, consumers will still not be able to compare food when purchasing online. Nutrition information does not automatically ensure healthy food choices, but it helps consumers to make an informed choice.

The WHO data platform is an important tool that has the ability to extensively improve the understanding and encourage regulation of the OOH food environment. The technology allows the collection and analysis of available data from MDAs. However, the current lack of available nutrition data is challenging.

Currently, the UK only requests companies with more than 250 employees to provide energy values of offered foods. Even though this is a good start, the regulation is not sufficient, and it might be easy for restaurants to find grey areas and loopholes to avoid providing energy information. More in-depth information, as well as standardized, complete, comparable, and transparent data, is needed.

The data platform showed that the majority of restaurants of four out of five cuisine types with the most restaurants on MDAs, which are pizza, breakfast, burgers, and chicken, and the vast majority of restaurants providing food, are independent restaurants. Only American cuisine has more chain restaurants. It is more likely that independent restaurants have fewer employers than chains. Therefore, the UK regulation might not apply to a large number of restaurants.

The presented data from the data platform show the high penetration of fast-food restaurants through MDAs. Even remote areas are within the delivery radius. In the example of England, the main chains tend to have products that are higher in calories on average and cover, with small exemptions, the entire country, making high-calorie products easily accessible.

Nutrient information is needed for consumer information, national regulations, and also marketing-related purposes. MDA data on sponsoring and marketing on the platform as well as the use of push notifications to nudge people into purchasing (more) food, as well as their effect, are relevant as they can influence people’s eating behavior. Regulations making sure healthy food options appear higher on lists or banning pictures of products high in energy might reduce the consumption of unhealthy foods.

Certain information can only be obtained with the support of the MDAs. Consumer-related information, providing insights into consumer behavior, would be essential to structure MDAs in a health-promoting way rather than in a way increasing overconsumption.

The data platform is an important tool to understand people’s eating behavior better as the digital food environment has a large role in populations’ diets and it monitors the environment on a continuous basis.

Due to inconsistent information, comparison and data analysis are currently still tricky. Some restaurants share calorie information per serving, others per 100 g or per portion. In some cases, no exact calorie information is provided or a calorie range is stated, including, e.g., different sizes or stuffing. In those cases, the average value is taken for analysis. Often this information is missing entirely.

## 5. Conclusions and Next Steps

The paper presented how this WHO platform can support closing the existing data gap in the digital food environment. The platform will be expanded to several countries and other meal delivery apps. In addition, developing an automated tool calculating average values of the most commonly purchased items or meal options is planned.

The platform has the potential to offer Member States a deep understanding of the digital food environment through MDAs. However, regulations are needed to enforce data sharing from MDA providers to public health researchers and governments.

Often the most vulnerable populations live in areas with high numbers of fast-food restaurants, serving food high in salt, fat, and sugar. Data gained from the data platform can help understand the number and distribution of restaurant types in certain areas as well as the average cost per calorie. This information can be used to compare restaurants with high-calorie options and less calorie-dense options and the average socioeconomic status of communities can be linked with the most popular restaurants in the area or by looking at the provision of meals in schools and the types of the most common restaurants in the area to tackle inequalities and to work on policies incentivizing healthy foods. Further, urban planning and healthy city design can be based on these data.

Analyses of topics like marketing on MDAs, understanding of the structure of MDAs, and the system behind the list of restaurants are needed. In some MDAs, options to filter the restaurants for “healthy” are available. This could support consumers making healthy food choices as long as the indicators labeling a food as healthy are clear. Otherwise, any kind of health claim can be included without any evidence. Due to the missing nutrition information, an accurate classification is not possible yet. The development of consistent food classification or food groups in MDAs can be helpful. Regulated portion sizes can also help consumers and especially families to avoid overeating.

If MDAs share their data and work together with the WHO, governments, and nutrition experts, they have a great potential to improve food choices. Policymakers can use the platform to argue for strong policies to protect consumers, share learnings, and compare data with other Member States but also to make MDA providers share essential consumer-related data to understand consumer behavior better. At the same time, food delivery services own extensive information about individual food behaviors of users, which would be essential for public health research to better understand food intake behaviors, influencing factors, and changes over time.

## Data Availability

Not applicable.

## Abbreviations

The following abbreviations are used in this manuscript:

AI	Artificial intelligence
EU	European Union
FOPL	Front of pack labeling
MDAs	Meal delivery apps
NCDs	Noncommunicable diseases
NPMs	Nutrient profile models
OOH	Out of home food environment
WHO	World Health Organization
YLDs	Years lived with disability

## Appendix A

The appendix shows the list of cuisine types including their average and maximum energy per menu item/kcal. It also indicates the number of chains per cuisine type.

**Cuisine Type**	**Energy per Menu Item/kcal**		**Number of** **Chains**
	**Average**	**Maximum**	
Cakes	719	9426	5
Carvery	657	4387	4
Desserts	380	9600	45
American	333	9600	71
Pub Food	319	1726	7
Peri Peri	315	7420	12
Latin American	310	2336	2
Burritos	300	6396	12
Mexican	275	6396	16
Chicken	269	7420	80
Sandwiches	256	4068	24
Pizza	246	3755	57
Breakfast	211	4068	34
Burgers	210	6660	102
Pasta	170	1557	8
Italian	167	2440	49
Coffee	159	4322	11
Steak	156	2363	6
Tapas	149	1544	2
Doughnuts	149	4250	4
Ice Cream	143	2314	10
Pies	139	2376	6
Lunch	139	4322	22
Sushi	127	4171	8
Lebanese	120	8203	5
Continental	115	1400	2
Vegan	114	4573	17
British	105	4387	12
Healthy	103	1960	15
BBQ	90	1638	5
Salads	88	1960	3
Mediterranean	78	8203	7
Cafe	77	3721	5
Noodles	75	2878	9
Middle Eastern	64	1330	4
French	64	2142	3
Bagels	62	667	2
Tex-Mex	62	1969	2
English	62	1849	4
Brunch	61	1174	2
Japanese	57	4171	13
Sweets	56	1267	6
Kebab	56	4943	9
Asian	55	2027	18
Street Food	52	3312	7
Local Legends	50	6660	24
Chinese	48	1223	17
Italian Pizza	45	1526	2
Smoothies	44	874	2
Fish & Chips	41	2024	7
Seafood	39	1500	2
Milkshakes	37	4560	12
European	34	2400	6
Roast Dinners	26	4387	2
Indian	19	1306	18
Bakery	17	4560	2
Bubble Tea	17	816	3
Korean	17	1068	9
Vegetarian	16	4560	4
Brazilian food	16	801	2
Waffles	14	1247	3
Sri Lankan	12	10,000	2
Fast Food	10	1271	6
Polish	9	2400	1
Groceries	6	1289	9
Alcohol	6	1289	9
Thai	5	1372	7
Grill	4	2440	7
